# *Hypericum lanceolatum *(Hypericaceae) as a potential source of new anti-malarial agents: a bioassay-guided fractionation of the stem bark

**DOI:** 10.1186/1475-2875-10-167

**Published:** 2011-06-17

**Authors:** Denis Zofou, Théodora K Kowa, Hippolyte K Wabo, Moses N Ngemenya, Pierre Tane, Vincent PK Titanji

**Affiliations:** 1 Biotechnology Unit, University of Buea. P.O. Box 63 Buea, South West Region, Cameroon; 2 Laboratory of Natural Product Chemistry, University of Dschang, P.O. Box 67 Dschang, West Region, Cameroon

## Abstract

**Background:**

Malaria is a major public health threat in Africa, and traditional medicine continues to play a key role in its control especially in rural areas. A bioassay-guided fractionation was carried out in order to evaluate the anti-malarial potential and the safety of the methanol extract of the *Hypericum lanceolatum *stem bark.

**Methods:**

The anti-plasmodial activity was assayed by the lactate dehydrogenase method (pLDH) against the multidrug-resistant W2mef laboratory strain, and a field isolate (SHF4) of *Plasmodium falciparum*. Cytotoxicity tests were carried out using the LLC-MK2 monkey kidney epithelial cells.

**Results:**

Five compounds were isolated from the most active and least cytotoxic ethylacetate sub-extract: betulinic acid (HLT1), 2,2',5,6'-tetrahydroxybenzophenone (HLT2), 5-hydroxy-3-methoxyxanthone (HLT3), 3-hydroxy-5-methoxyxanthone (HLT4) and HLT0 (yet to be identified). Three of the tested compounds presented significant anti-plasmodial activities (with 50% inhibitory concentration, IC_50 _< 5 μM), with 5-hydroxy-3-methoxyxanthone exerting the highest activity, followed by HLT0 and betulinic acid. All the compounds with significant anti-plasmodial activity were non-cytotoxic, except betulinic acid which showed a 50% cytotoxic concentration, CC_50 _of 25 μg/mL.

**Conclusions:**

These findings justify the use of *H. lanceolatum *stem bark as anti-malarial by traditional healers of Western Cameroon, and could constitute a good basis for further studies towards development of new drug candidates or phytomedicines for malaria.

## Background

The fight against malaria was identified by the United Nations in the Millennium Development Goals as an objective and remains one of the priorities for the World Health Organization. Chemotherapy is central to any strategy for effective reduction of mortality related to malaria, since an efficient vaccine is yet to be approved [1]. The emergence and relentless spread of resistance against all the drugs in current use, including the newly introduced artemisinin-based combination therapy, have aggravated the disease burden in endemic regions [2,3]. Hence, there is an urgent need to discover new efficacious and safe anti-malarial drugs in order to face this situation.

For decades, traditional herbal medicine had constituted a good basis for anti-malarial lead discovery and drug development. A typical example is quinine, which was the first anti-malarial drug of plant source, isolated from the bark of *Cinchona *tree (Rubiaceae) in 1820 [4]. Quinine is one of the oldest and most important anti-malarial drugs, which remains a first line drug today for the treatment of severe malaria. This molecule also served as template for the synthesis of prominent chloroquine and mefloquine [5]. More recently, artemisinin isolated from the Chinese plant *Artemisia annua, *has been used successfully against multidrug-resistant strains of malaria parasites [6]. Previous investigations showed that over 200 plants species were identified in Cameroon for their use in malaria treatment by traditional healers, and some of these were proven to contain active ingredients with significant anti-plasmodial activity [7-9]. *Hypericum lanceolatum *also known as *Hypericum revolutum *subsp. *revolutum, Norysca lanceolata, Campylosporus angustifolius*, *Campylosporus madagascariensis, Campylosporus reticulatus, Hypericum angustifolium, Hypericum madagascariense*, *Hypericum revolutum*, is a multipurpose plant, commonly used in Cameroon traditionally to treat several ailments including malaria, skin infections, venereal diseases, gastrointestinal disorders, tumours, infertility and epilepsies [10]. The stem bark is usually boiled in water and administered either as steam bath or orally for the treatment of malaria and other fevers. The roots are also known for their use against intestinal worms and dysentery. They are combined with *Mangifera indica *leaves, boiled and administered as a drink. In the Lebialem Division (South West Region), decoction of fresh leaves is taken orally to treat nerves problems [10].

However, despite the endemic use of *H. lanceolatum *as an anti-malarial herbal medicine in Cameroon, its efficacy is yet to be experimentally established. A bioassay-guided fractionation of the stem bark of this plant was conducted in order to assess the *in vitro *anti-plasmodial activity, thus the potential of the plant species as source of new malaria drug leads.

## Methods

### Collection of plant materials

Stem bark of *H. lanceolatum *was collected on the Mount Bamboutos flanks (Western Region, Cameroon) in May 2009. The sample identification was confirmed by Mr. Victor Nana, botanist at the Cameroon National Herbarium in Yaounde, where a voucher specimen was deposited (Voucher No 32356/HNC).

### Preparation of crude extract

The air-dried and powdered plant material (1.5 Kg) was macerated for three days at room temperature in 5 L of methanol, filtered with Whatman paper, and the solvent evaporated using a Rotavapor system (BÜCHI Labortechnik AG, Switzerland) to obtain a crude extract (170 g) which was stored at - 20°C till further use.

### Bioassay-guided fractionation, isolation and characterization of pure compounds

The fractionation as well as the purification and characterization of isolated compounds were done as previously described by Tane *et al *[11]. Only extracts or fractions with IC_50 _< 10 μg/mL and CC_50 _> 30 μg/mL were considered for the further steps. An amount of 150 g of the MeOH extract was dissolved in 1 L of distilled water and sequentially partitioned with *n*-hexane (5 L), EtOAc (5 L), and *n*-BuOH (5 L) to give respective extracts. All the three sub-extracts were screened for their anti-plasmodial and cytotoxicity profiles and the EtOAc sub-extract was retained for further investigation provided its high activity and relative safety. An amount of 45 g of this extract was subjected to a silica gel column chromatography eluted with gradients of *n*-hexane-EtOAc (10:0; 9:1; 8:2; 1:1; 0:10) and EtOAc-MeOH (10:0; 9:1; 8:2; 1:1; 0:10). Twenty seven fractions of 500 mL each were collected and combined on the basis of their TLC profiles into five major fractions F1-F5 (F1: 1-5; F2: 6-7; F3: 8-12; F4: 13-21; F5: 22-27). Fraction F1 (3 g) contained mostly mixture of sterols and was not considered for further investigation given the poor outcome of the biological screening. Fraction F2 (4.5 g) was purified on a silica gel column chromatography (50 mm × 600 mm), eluted with a gradient of *n*-hexane-EtOAc (100:0; 95:5; 90:10; 85:15; 80:20; 0:100), to give HLT1 (90 mg, Rf = 0.48; n-hexane-EtOAc 80:20) and HLT2 (150 mg, Rf = 0.5; n-hexane-EtOAc 95:5). Fraction F3 (10.6 g) was submitted to a silica gel column chromatography (50 mm × 600 mm), eluted with gradients of CH_2_Cl_2_-EtOAc (100:0; 95:5; 90:10; 85:15; 80:20; 70:30, 0:100), and EtOAc-MeOH (100:0; 95:5; 90:10), to yield HLT3 (9 mg, Rf = 0.28; n-hexane-EtOAc 60:40) and HLT0 (8 mg, Rf = 0.32; n-hexane-EtOAc 60:40). Fraction F4 (12.8 g) was chromatographed on a silica gel column with *n*-hexane-EtOAc (100:0; 95:5; 90:10; 85:15; 80:20; 0:100) and EtOAc-MeOH (100:0; 95:5; 90:10; 85:15; 80:20; 70:30, 0:100). Sub-fractions eluted by *n*-hexane-EtOAc (8:2) were further purified on Sephadex LH-20 using CH_2_Cl_2_-MeOH (1:1) to give HLT4 (30 mg, Rf = 0.40; n-hexane-EtOAc 60:40).

The structures of the isolated compounds were elucidated using spectroscopic methods and comparison done with published data. Melting points (uncorr.) were recorded on a Reichter microscope or on a Büchi melting point B-540 apparatus. The IR and UV spectra were recorded on a Shimadzu FTIR-84000S spectrophotometer and a Jasco V-550 UV/Vis spectrophotometer, respectively. ^1^H NMR and ^13^C NMR spectra were recorded on Joel JNM ECA-600 and AL-400 spectrometers. Chemical shifts (*δ*) were reported in parts per million (ppm) with the residual solvent signals as internal reference. Coupling constants (*J *values) were given in Hertz. Column chromatography was run with Merck silica gel 60 and Sephadex LH-20. Analytical TLC was carried out on silica gel (Merck GF_254_) precoated plates with spots detected with an UV lamp at 254 and 366 nm and further visualized by spraying with 50% H_2_SO_4_, followed by heating at 100°C.

### Parasite strain

The W2mef (MRA-615) strain was ordered from the Malaria Research and Reference Reagent Resource Centre (MR4, Manassas, VA, USA), and maintained in continuous culture. The SHF4 field isolate previously isolated from a malaria patient in Buea in the South West region of Cameroon, had been stored in liquid nitrogen at the Biotechnology Unit, University of Buea.

### *Plasmodium falciparum *culture and maintenance

The field isolate and the laboratory strain of *P. falciparum *were grown and maintained in culture using the method of Trager and Jensen with some modification [8,9,12]. All the chemicals except Albumax II (Gibco; Invitrogen, USA), were ordered from Sigma-Aldrich Inc (Germany). Cultures consisted of a 4% haematocrit suspension of O+ human erythrocytes in RPMI 1640 medium supplemented with a gentamicin solution at 0.01 mg/mL, 25 mM HEPES buffer, 25 mM NaHCO_3_, and 1% Albumax II. Cultures were fed with a gas mixture consisting of 5% CO_2 _and incubated at 37°C (CO_2 _incubator Heraeus, Hera cell 150; USA). The estimation of the parasitaemia as well as parasite visualization before incubation was done using both fluorescence (Acridine Orange) and normal light (Giemsa stain) microscopes.

### Determination of *in vitro *anti-plasmodial activity

The drug sensitivity assay was carried out in 96-well microtitration plates as described by Desjardins *et al *with some modifications [8,13]. All stock solutions were sterilized by passing them through a 0.2 μm syringe filter and stored at -20°C until required. Similarly, a 2 μg/mL stock solution of quinine dihydrochloride (*Rotexmedica*, Trittau, Germany) was prepared and used as positive control. The parasitaemia was measured using the parasite lactate dehydrogenase (pLDH) assay.

### Analysis of test results from the pLDH assay

The pLDH assay generates optical density (OD) values at various concentrations of the drug as raw data. OD values from control wells represent the maximum amount of pLDH produced by parasites and OD values from blank wells (containing a 4% suspension of non parasitized red blood cells in malaria culture medium) represented background pLDH activity. A 100% growth value, which corresponds to maximum LDH activity, was obtained by subtracting the mean OD value of blank wells from that of control wells. Likewise, the growth value at each concentration of the drug was obtained by adjusting OD values from drug-treated wells for background pLDH activity (parasite-free red blood cells). These values were then expressed as a percentage of the maximal growth value and plotted against corresponding concentrations of the drug using the software HN-NonLin V1.1 [14] to generate log dose-response curves from which the 50%, 90%, 95% and 99% Inhibitory Concentrations (IC_50_, IC_90_, IC_95 _and IC_99 _respectively) were obtained. Each product was tested in triplicate in each of the two separate experiments, giving a total of six repeats per product and per concentration tested. The IC_50 _values obtained from these replicates were pooled and expressed as geometric mean IC_50 _values and the different means compared among themselves by independent samples t-test using SPSS Statistics 17.0 (Chicago, USA).

### Cytotoxicity study of active compounds and extracts

The cytotoxicity profiles of the extracts and pure compounds were estimated against LLC-MK2 monkey kidney epithelial cells according to the procedure previously described [14] with some modifications [9]. Cells were cultured under the same conditions as *P. falciparum*. Cells were distributed in 96-well plates at 20,000 cells per well in 100 μL culture medium, and allowed to attach and become confluent, for 24h. The medium was then removed completely the next day and 100 μL of fresh medium was added to all the wells, followed by 100 μL of crude extract (1000 μg/mL) or pure compounds (200 μg/mL) added in triplicate in row H. A two folds serial dilution was made upward to obtain concentration range of 250 - 3.90 μg/ml (crude extract) or 50 - 0.78 μg/ml (pure compounds), cells at row A serving as control without drug. The plates were incubated for five days at 37°C in 5% CO_2 _in air. Cells concentration and viability in the presence of extracts or pure compounds were compared with that of control cultures without extracts. The definition of the cytotoxicity used [15] was: CC_50 _< 1.0 μg/ml (high cytotoxicity); CC_50 _1.0 -10.0 μg/ml (moderate); CC_50 _10.0-30.0 μg/ml (mild); and CC_50 _> 30 μg/ml (non toxic). The selectivity index defined as SI = CC_50_/IC_50 _was also considered with a product considered cytotoxic when SI < 10 [16].

## Results

### Anti-plasmodial activity and cytotoxicity of sub-extracts and fractions from *H. lanceolatum*

The anti-plasmodial activity and cytotoxicity profiles of the sub-extracts and fractions from *H. lanceolatum *extract are summarized in Table 1. The ethylacetate sub-extract showed a good activity (IC_50 _< 10 μg/mL) against W2mef strain of *P. falciparum*, while the two other sub-extracts exhibited weaker activities. None of the three sub-extracts was significantly cytotoxic (CC_50 _> 30 μg/mL).

**Table 1:** Anti-plasmodial activity and cytotoxicity of sub-extracts and fractions from *H. lanceolatum*

Sub-extract or Fraction	IC_50 _on W2mef (μg/mL)	CC_50 _on LLC-MK2 cells (μg/mL)	Selectivity Index (SI)
**MeOH crude extract**	3.98 ± 0.11	> 1000	-
**Ethylacetate sub-extract**	5.02 ± 1.01	204.86 ± 2.00	40.80 ± 0.39
**Aqueous sub-extract**	15.48 ± 6.25	125 ± 0.00	8.07 ± 0.00
***n*-butanol extract**	11.95 ± 5.81	>1000	-
**F1**	15.60 ± 1.35	46.87 ± 15.62	3.00 ± 1.00
**F2**	6.37 ± 2.17	125 ± 0.00	19.62 ± 0.00
**F3**	3.91 ± 0.01	250 ± 0.00	39.24 ± 0.00
**F4**	5.33 ± 2.47	31.25 ± 0.00	5.86 ± 0.00
**F5**	14.52 ± 0.46	>1000	-
**QN**	**0.27 ± 0.04**	-	-

QN: Quinine

The fractionation of the most active ethylacetate sub-extract yielded five major fractions that were also screened against W2mef, with F3 exhibiting the highest activity, followed by F4, F2; whereas fractions F1 and F5 showed weak activity. Except fraction F4 which with a CC_50 _slightly below the 30 μg/mL cut off point, none of the fractions showed significant cytotoxicity.

### Description of the compounds isolated from *Hypericum lanceolatum*

**Figure 1:**
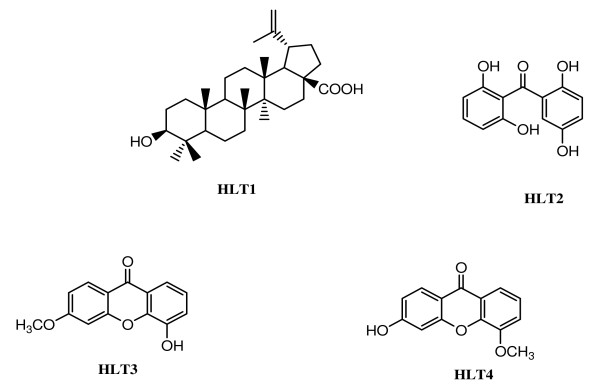
**Structure of the compounds isolated from the ethylacetate sub-extract of *H. lanceolatum *stem bark**.

The structures of the compounds isolated are shown in the Figure 1. The purification of fraction F2 led to isolation of two compounds, HLT1 and HLT2. Compound HLT1 was obtained as colourless needles from hexane-EtOAc, with a melting point of 319-320°C. It reacted positively to the Liebermann-Burchard test, which is characteristic of triterpenes. Its molecular formula was determined as C_30_H_48_O_3 _and the molecular weight was 456. HLT2 was obtained as yellow needles and had a melting point of 201-202°C. It reacted positively to FeCl_3 _reagent, suggesting the presence of phenolic hydroxyl group in the molecule. Its molecular formula C_13_H_10_O_5_, corresponding to a molecular weight of 246.

Fraction F3 led to the isolation of HLT0 and HLT3. Compound HLT0 appears as yellow powder. HLT3 was equally obtained as yellowish powder having 166-168°C as melting point. It reacted positively to FeCl_3 _reagent, suggesting the presence of phenolic hydroxyl group in the molecule. The molecular formula was determined as C_14_H_10_O_4 _with a molecular weight of 242. Purification of fraction F4 yielded one compound codified as HLT4. This appeared as a yellowish powder from CH_2_Cl_2_, with melting point 284-285°C. It reacted positively to FeCl_3 _reagent, suggesting the presence of phenol-hydroxyl group in its structure. The molecular formula was determined as C_14_H_10_O_4 _and the molecular weight was 242.

### Extraction yield and anti-plasmodial activity of the pure compounds of *Hypericum lanceolatum*

The extraction yield and anti-plasmodial activity of compounds isolated from the ethylacetate sub-extract of *H. lanceolatum *are presented in Table 2. Among the five compounds isolated, HLT2 had the highest extraction yield (3.33% of the dry fraction), followed by HLT1 whereas HLT3 showed the lowest yield (0.08%). Three of the five compounds tested showed good anti-plasmodial activity (HLT0, HLT1 and HLT3) against the multidrug-resistant W2mef parasite strain, with IC_50 _values below 5 μg/mL; all the remaining ones being weakly active (IC_50 _> 10 μg/mL) on both W2-mef and field isolate SHF4.

**Table 2:** Extraction yield and anti-plasmodial activity of pure compounds isolated from *H. lanceolatum*

Compound (code)	Quantity (mg)	Extraction yield	IC_50 _on W2mef (μM)	IC_50 _on SHF4 (μM)
**HLT0**	8	0.07	4.26 ± 0.15*	5.89 ± 0.20*
**Betulinic acid (HLT1)**	90	2.0	4.50 ± 1.35	5.60 ± 0.13
**2,2',5,6'-Tetrahydroxybenzophenone (HLT2)**	150	3.33	55.12 ± 0.93	13.41 ± 0.16
**5-Hydroxy-3-methoxyxanthone (HLT3)**	9	0.06	3.26 ± 0.08	1.43 ± 0.48
**3-Hydroxy-5-methoxyxanthone (HLT4)**	30	0.11	33.84 ± 0.20	34.09 ± 0.12
**QN**	-	-	**0.27 ± 0.04**	**0.14 ± 0.05**

*IC_50 _expressed in μg/mL; Extraction yield expressed in % weight/weight of the ethylacetate fraction

### Cytotoxicity of the tested products on LLC-MK2 monkey kidney epithelial cells

Table 3 presents the result of the cytotoxicity of the tested compounds against the LLC-MK2 cells. Two out of the 5 compounds were significantly cytotoxic (CC_50 _> 30 μg/mL) while one presented a low toxicity and the remaining three were non toxic.

**Table 3:** Cytotoxicity profile of *H. Lanceolatum *compounds

Compound (code)	CC_50 _on LLC-MK2 (μg/mL)	Mean SI for W2mef	Mean SI for W2mef
**HLT0**	37.50 ± 6.25	7.48	6.27
**Betulinic acid (HLT1)**	25.00 ± 0.00	4.98	4.46
**2,2',5,6'-Tetrahydroxybenzophenone (HLT2)**	> 100	-	-
**5-Hydroxy-3-methoxyxanthone (HLT3)**	> 100	-	-
**3-Hydroxy-5-methoxyxanthone (HLT4)**	12.50 ± 0.00	0.36 ± 0.00	0.36

Cytotoxicity was categorised as follows [15]: CC_50 _< 1.0 μg/ml (high cytotoxicity); CC_50 _1.0 -10.0 μg/ml (moderate); CC_50 _10.0-30.0 μg/ml (mild); and CC_50 _> 30 μg/ml (non toxic).

## Discussion

From the MeOH crude extract prepared from the stem bark of *H. lanceolatum*, three sub-extracts were obtained and screened both for anti-plasmodial activity against the multidrug-resistant W2mef strain of *P. falciparum*, and cytotoxicity on the LLC-MK2 monkey kidney epithelial cells. The ethylacetate sub-extract was shown to be the most active, followed by the *n-*butanol whereas the water sub-extract was least active. In its traditional use in Western Cameroon, the stem bark is boiled in water and administered as a tea, either alone or in combination with other plants. The low activity of the water soluble portion of the extract therefore shows that the use of water as solvent may not be appropriate for *H. lanceolatum *stem bark in the treatment of malaria. This finding instead predicts the low polarity of the active ingredients of the plant part. The activities of the different sub-extracts were lower than the one of the main methanol crude extract; this observation may be an indication of synergistic interactions among the compounds found in the main extract.

The fractionation of the most active sub-extract led to five main fractions among which three were retained for further purification based on their anti-malarial potential and relative safety. The purification of the three active fractions yielded five compounds. Four of these compounds are well-known and two of them had been isolated from other plant species, while two were previously obtained by synthesis; none of them was known before as occurring in *H. lanceolatum*.

From its spectral data, HLT1 was identified to be 3-hydroxy-lup-20(29)-en-28-oic acid commonly known as betulinic acid, a naturally occurring pentacyclic triterpenoid found in the bark of several plants species, principally the white birch (*Betula pubescens*) from which its name derives, but it was also isolated from *Lycopus lucidus*, *Ziziphus mauritiana*, *Prunella vulgaris*, the tropical carnivorous plants *Triphyophyllum peltatum *and *Ancistrocladus heyneanus, Diospyros leucomelas, Tetracera boiviniana*, the jambul *Syzygium formosanum *[17,18]. Betulinic acid was also directly synthesized from betulin, which is an abundant compound of white birch, *Betula alba *[19].

HLT2 was identified as 2,2',5,6'-tetrahydroxybenzophenone, a compound that was previously synthesized in 1982 by Suzuki et collaborators from Matsushita Electric Industrial Co., Ltd., an Osaka-based Japanese company, and registered under the USA Patent number 4425404 [20]. Similarly, HLT3 identified as 5-hydroxy-3-methoxyxanthone was described by Pedro *et al *in 2002 [21]. HLT4 which was identified to be the 3-hydroxy-5-methoxyxanthone has equally been synthesized from a natural xanthone template by Gnerre *et al *[22].

The anti-plasmodial activities of the isolated products were 3.26 - 55.12 μM against multidrug-resistant W2mef laboratory strain, and 1.43 - 34.09 μM against the SHF4 field isolate, with three compounds having good activity. These are 5-hydroxy-3-methoxyxanthone, betulinic acid and HLT0.

5-Hydroxy-3-methoxy-xanthone (HLT3) scored the highest *in vitro *activity among the compounds tested. Several xanthones of plant source have been reported for their anti-malarial activity. The ethanol extract of the bark of *Garcinia dulcis *(Guttiferae) furnished five xanthones among which garciniaxanthone showed the highest inhibitory effects on the growth of *P. falciparum *with IC_50 _value of 0.96 μg/mL [23]. Anti-malarial xanthones were equally isolated from *Vriesea sanguinolent *leaves *(*6-hydroxyluteolin-7-*O*-(1^2^-*a*-rhamnoside, IC_50 _= 2.13 μg/mL against K1) and *Andira inermis *leaves and stem bark (IC_50 _= 4.1 μg/mL against Dd2) [5]. However, the anti-plasmodial activity of 5-hydroxy-3-methoxyxanthone has not been documented. The isomer of this compound, 3-hydroxy-5-methoxyxanthone, also isolated from the same sub-extract of *H. lanceolatum *stem bark, was inactive both against W2mef and the field isolate of the malaria parasite. This observation suggests a key role played by the 5-hydroxyl group and/or 3-methoxyl group in the mechanism of action of the molecule. Further investigations including structure-activity relationship study are necessary for a full understanding of the mechanism of action of the 5-hydroxy-3-methoxyxanthone, and optimization of this hit into a malaria drug candidate.

Betulinic acid is a multipotential molecule with anti-inflammatory, anti-tumour, anti-angiogenesis, anti-neoplastic [24-27] and anti-viral anti-HIV activities [28,29]. The anti-plasmodial potential of this molecule was equally established by previous studies. The molecule isolated from the root bark of the Tanzanian species *Uapaca nitida *was screened for its *in vitro *anti-plasmodial activity of against CQ-resistant K1 and CQ-sensitive T9-96 *P. falciparum *and exhibited a weak activity against both strains, with IC_50 _of 19.6 and 25.9 μg/mL, respectively [30,31]. Betulinic acid was equally isolated from the Cameroonian plant *Psorospermum glaberrimum *and tested for its activity against the W2 strain of *P. falciparum*, with an IC_50 _of 5.10 μM which is similar to the value of 4.50 μM obtained in this study with the W2mef clone. These results, therefore, confirm the anti-plasmodial potential of this molecule [32].

The methanol crude extract of *H. lanceolatum *showed no sign of cytotoxicity against LLC-MK2 cells, even at 250 μg/mL. However, the aqueous sub-extract deriving from this was slightly cytotoxic at high concentrations, although the toxicity level was not considerable (CC_50 _> 30 μg/mL). Out of the five compounds isolated and tested, only betulinic acid and 3-hydroxy-5-methoxyxanthone were mildly cytotoxic (10 ≤ CC_50 _≤ 30 μg/mL). Zuco *et al *[17] studied the *in vitro *cytotoxicity of betulinic acid in melanoma and non-melanoma tumour cell lines. Betulinic acid exerted its anti-proliferative activity on all the tested lines in a very narrow range of doses (1.5 - 4.5 mg/mL), and was effective against wild-type p53 and mutant p53 neoplastic cell lines derived from cancers clinically resistant to conventional anti-neoplastic drugs. However, the activity observed was selective of tumour cells, as the compound showed no significant cytotoxic activity against neural cells *in vitro *[17,33]. Cytotoxicity of several xanthones has been fully documented [34,35]. The present findings therefore corroborate the previous results.

In conclusion, the study shows that the ethylacetate extract of *H. lanceolaum *stem bark has a higher anti-malarial activity and least cytotoxicity, compared to the *n*-butanol and water extracts. The use of medium polar solvent in the preparation of natural medicine is therefore recommended. This extract contains ingredients with different levels of anti-plasmodial activity, 5-hydroxy-3-methoxyxanthone, the known betulinic acid and HLT0 being the most prominent compounds. These findings therefore justify the use of stem bark of *H. lanceolatum *in Cameroonian folk medicine for the treatment of malaria. Further work including *in vivo *anti-malarial and toxicity testing, are likely to yield new anti-malarial drug candidates. Equally, the standardization of the extracts could lead to effective phytomedicines which could be locally exploited for the good of the populations who may be unable to afford standard pharmaceutical drugs.

## List of abbreviations

IC_50_: 50% inhibitory concentration; CC_50_: 50% cytotoxic concentration; MeOH: methanol; EtOAc: ethylacetate; *n*-BuOH: *n*-butanol; CH_2_Cl_2_: methylene chloride.

## Competing interests

The authors declare that they have no competing interests.

## Authors' contributions

DZ contributed in running the laboratory work, analysis of the data, and drafted the paper. TKK contributed in collecting plant samples, preparing the crude extracts, and purifying compounds. HKW contributed in compounds purification and structure determination. MNN contributed in the biological studies. PT designed the chemistry part of the work and contributed in chromatographic analysis. VPKT conceived the entire project and supervised it all through. All the authors have read the final manuscript and approved the submission.
